# Editorial: Role of neuroimaging in the diagnosis and treatment of rare diseases

**DOI:** 10.3389/fnimg.2025.1566484

**Published:** 2025-02-13

**Authors:** Mohammed Salman Shazeeb, Maria T. Acosta, Cynthia J. Tifft

**Affiliations:** ^1^Image Processing and Analysis Core (iPAC), Department of Radiology, University of Massachusetts Chan Medical School, Worcester, MA, United States; ^2^National Human Genome Research Institute, National Institutes of Health, Bethesda, MD, United States

**Keywords:** neuroimaging biomarker, GM1 gangliosidosis, idiopathic dystonia, pediatric brain tumor, birth nonprogressive neuromuscular disease

## 1 Introduction

Rare diseases are individually rare, affecting <5 in 10,000 people worldwide, yet collectively common, affecting 6%−8% of the human population (Dawkins et al., 2018). An estimated 6,000–8,000 disease entities have been described with the numbers rising steadily with new diagnostic techniques such as exome, genome, and RNA sequencing. The diagnostic odyssey can be lengthy, affecting quality of life for patients and their caregivers and can lead to reduced life-expectancy. Definitive treatment for most rare diseases is lacking although progress is being made. Many rare disorders affect the central nervous system (CNS) and advanced neuroimaging modalities like magnetic resonance imaging (MRI) have been shown to aid both in diagnosis and in monitoring patient outcomes in clinical trials. In this Research Topic, we highlight advances made in the diagnosis and/or treatment of rare diseases using MRI neuroimaging biomarkers. The diseases include type II GM1 gangliosidosis, idiopathic dystonia, pediatric brain tumors including pediatric posterior fossa brain tumor and diffuse midline glioma of the pons, and birth nonprogressive neuromuscular diseases.

## 2 Neuroimaging biomarkers in rare diseases

### 2.1 Type II GM1 gangliosidosis

Type II GM1 gangliosidosis (GM1) is an ultra-rare autosomal recessive disorder of ganglioside degradation with an incidence of 1:100,000–200,000 live births (Hoskins, 2022; Caciotti et al., 2011). Deficiency of lysosomal hydrolase b-galactosidase impairs the degradation of GM1 ganglioside, a normal component of the neuronal membrane, leading to demyelination, neuronal loss and progressive brain atrophy. The disorder is uniformly fatal with no approved therapies. Since the neurodegenerative changes and atrophy in GM1 patients are severe, automated methods are yet to be developed to appropriately register GM1 brains to conventional brain atlases to perform automated segmentations. The study by Zoppo et al. proposed a standardized method to perform brain MRI volumetric measurements in GM1 patients. The volumetric analysis (using manual and semi-automated segmentations of small and large brain structures, respectively) proposed in this study established good inter- and intra-rater reliability in measuring the brain structures to characterize the late-infantile and juvenile subtypes of GM1. With the emergence of gene therapy to treat GM1 patients, brain volumetrics can serve as a useful neuroimaging biomarker to track the state of the disease and response to treatment.

### 2.2 Idiopathic dystonia

Dystonia is a rare neurological disease characterized by involuntary muscle contractions leading to abnormal posture and movements. Classification of the disease based on distinct symptom distribution is clinically important and widely used for different implications of therapy (Albanese et al., 2013). Cervical dystonia (CD) and generalized dystonia (GD) are two common subtypes. While the premier subtype is a typical focal form involving exclusive neck twisting/abnormal posture, the latter mainly affects the trunk and even wider body regions. The global prevalence of idiopathic dystonia is estimated to be around 16–30 per 100,000 (Medina et al., 2022). Since idiopathic dystonia begins without certain causes or positive findings in conventional imaging scans, it is challenging to trace the abnormal brain structures or circuits involved in the mechanism of the disease. Wu et al. investigated volumetric and morphometric changes in the cortical and subcortical regions of the brains of patients with CD and GD. They demonstrated that cortical thickness abnormalities in different brain regions and reduced subcortical volumes with morphometric changes indicated disease progression. Moreover, clinical associations with volumetric/morphometric changes correlated with disease severity of CD and GD and can serve as neuroimaging biomarkers in tracking disease progression and response to potential treatments.

### 2.3 Pediatric brain tumors

Brain tumors are the leading cause of pediatric cancer deaths in the United States. In children between the ages of 0–19 years the incidence of brain and other CNS tumors between 2014–2018 was 6.23 per 100,000 (Ostrom et al., 2018). From these tumor occurrences, over half appear in the posterior fossa and can be treated with surgical resection, chemotherapy, and irradiation. However, diffuse intrinsic pontine glioma (DIPG) are highly aggressive brain tumors that carry a poor prognosis due to treatment difficulty. Tanedo et al. implemented diffusion tensor imaging (DTI) using fractional anisotropy maps to visualize the impact of surgery and chemotherapy on microstructural changes in white matter tracts in pediatric posterior fossa brain tumor survivors. The white matter changes can potentially help understand neurocognitive functioning in these patients as they grow into adulthood. Another study by Szychot et al. used imaging to assess the distribution of infusate in children with DIPG treated with convection-enhanced delivery (CED) of therapeutic drugs. They proposed that volumetric T2-weighted MRI in combination with the apparent diffusion coefficient from diffusion-weighted imaging can provide a reliable method to evaluate CED infusate distribution. These neuroimaging biomarkers could prove valuable in future clinical trials of CED infusions within brain tissue, where accurate anatomical localization of infusate distribution will be essential for demonstrating treatment efficacy.

### 2.4 Birth nonprogressive neuromuscular disease

Birth nonprogressive neuromuscular disease refers to motor deficits observed in newborns that can be caused by congenital disease or obstetrical trauma. Examples of this disease include arthrogryposis multiplex congenita (AMC) and obstetrical brachial plexus palsy (OBPP). The incidence of AMC and OBPP is reported to be around 0.03–0.1 and 0.4–4 per 1,000 live births, respectively (Tolmacheva et al.). The mini-review article presented by Tolmacheva et al. highlights the importance of brain mapping in this patient population by presenting several studies that use functional MRI (fMRI) to investigate cortical activation to assess motor performance. Quantification of cortical plasticity using fMRI can be an important neuroimaging biomarker to personalize effective neuromodulation therapy that can potentially help with functional motor recovery.

## 3 Conclusion

This Research Topic presents an array of rare neurologic disorders and demonstrates the utility of neuroimaging to assess and monitor the state of disease. In some cases, the authors demonstrate how neuroimaging can also be used as a biomarker to assess and monitor the effects of therapeutic interventions. With recent advances in new techniques such as gene therapy, there is tremendous potential to develop treatments for rare diseases and neuroimaging biomarkers can play a vital role not only in diagnosing and characterizing the disease, but also in tracking response to therapy and improvements in overall wellbeing.
